# Increased B cell deletion and significantly reduced auto-antibody titre due to premature expression of human complement receptor 2 (CR2, CD21)

**DOI:** 10.1016/j.molimm.2008.08.273

**Published:** 2009-03

**Authors:** Isabel Y. Pappworth, Liudmila Kulik, Catherine Haluszczak, Jason W. Reuter, V. Michael Holers, Kevin J. Marchbank

**Affiliations:** a Institute of Human Genetics, Newcastle University, Central Parkway, Center for Life, Newcastle-upon-Tyne NE1 3BZ, UK; b Department Medicine and Immunology, University of Colorado Denver, School of Medicine, Aurora, CO 80045, USA

**Keywords:** Transgenic/knockout, Complement, B lymphocytes, Auto-antibodies, Apoptosis

## Abstract

The involvement of complement receptor 2 (CR2) in B cell tolerance and autoimmune disease has been revealed over the past decade or so. Our previous studies have established that mice prematurely expressing human CR2 under the control of a lambda light chain promoter (in particular the hCR2^high^ line) have a marked deficit in their immune response to various antigens and fail to develop collagen-induced arthritis. This phenotype appears to be the result of irreversible changes in B cell signalling pathways and suggested that hCR2 expressing mice are protected from developing autoimmune disease. To test this hypothesis, we examined the ability of the hCR2 to block the development of spontaneous autoimmune disease on the C57BL/6j-Fas^lpr/^Fas^lpr^ (B6^lpr^) background. We found that expression of hCR2 on the B6^lpr^ background resulted in a significant reduction in levels of anti-nuclear antibodies (ANA) generated as mice aged but the levels of ANA were still higher than those found in age matched C57BL/6j (B6) mice. B cells from hCR2^high^ mice were found to display a higher baseline level of apoptosis, whether analysed *ex vivo* or after *in vitro* culture, than their B6 counterparts and this was apparently linked to both surface IgM expression by the B cells and C3 levels in the mice. Our data also provides evidence that B cell survival in the presence of hCR2 is heavily modified by the background strain of the mouse. Overall, we have demonstrated that mice expressing hCR2 on their B cells during bone marrow development display a higher degree of apoptosis which may lead to a deletion of autoreactive B cells and be protective against the development of autoimmune disease.

## 1 Introduction

The suggestion that complement receptor type 2 (CR2) expression levels might be important in autoimmune disease arose from two studies that noted a marked down regulation of CR2 expression levels on B lymphocytes isolated from patients with systemic lupus erythematosus (SLE) (Marquart et al., 1995; Wilson et al., 1986). The reduction in CR2 expression levels on B cells as disease progressed in a murine model of SLE (the MRL^lpr^ model) further supported this assertion (Takahashi et al., 1997). Indeed, data generated using mice deficient in the *Cr2* gene (encoding both mouse CR1 and CR2 through alternative splicing; *Cr2*^−/−^ (Kurtz et al., 1990; Molina et al., 1990)), appeared to confirm that both these proteins were important in the development of auto-antibodies in lupus susceptible mice (Wu et al., 2002). However, probably the most compelling evidence to link CR2 function to the onset of lupus arose from a study of lupus susceptibility loci in the NZM2410 mice (Boackle et al., 2001). This study identified a genetic polymorphism that results in the addition of a novel glycosylation site on the CR2 protein. This interferes with binding to its main ligand, C3d, and supported previous work suggesting a necessity for B cell signalling through CR2 in maintaining B cell tolerance (Carroll, 2000; Fischer et al., 1996; Prodeus et al., 1998). A genetic polymorphism in the human CR2 promoter region (that directly influenced CR2 expression levels) was recently identified in a cohort of SLE patients (Wu et al., 2007), again suggesting that CR2 expression level and/or functionality was highly important in overall B cell function.

CR2 is expressed primarily on mature B cells and follicular dendritic cells (FDC) in both human and mouse. hCR2 and mCR2 have a high degree of homology both at the nucleotide and protein level being approximately 150 kDa in size and consisting of an extracellular domain, made of 15 or 16 tandem short consensus repeats (SCR), a transmembrane domain and a short intracellular domain (Fingeroth, 1990; Molina et al., 1990; Moore et al., 1987; Weis et al., 1988). However, one important difference between these two species is that hCR2 and hCR1 are the products of two separate genes whilst mCR1 and mCR2 are the products of one alternatively spliced gene (*Cr2*) (Ahearn and Fearon, 1989). In the normal situation, expression of CR2 on human and mouse B lymphocytes is restricted to transitional B cells and mature B cells as well as marginal zone and B-1 B cells (Takahashi et al., 1997; Tedder et al., 1984). B cells at earlier stages of development do not express CR2. The role for CR2 in the development and maintenance of the humoral response to T-dependent (TD) antigens (Ags) was first discovered by several *in vivo* studies using CR1/2 blocking Abs and a study using CR2-IgG fusion protein (Gustavsson et al., 1995; Hebell et al., 1991; Heyman et al., 1990; Thyphronitis et al., 1991). This role was confirmed and expanded by the independent generation, by gene targeting, of 3 lines of *Cr2*^−/−^ mice (Ahearn et al., 1996; Haas et al., 2002; Molina et al., 1996). *Cr2*^−/−^ mice have been shown to have a defect in response to T-independent (TI) Ag (Haas et al., 2002) and to T-dependent (TD) antigens (Ahearn et al., 1996; Molina et al., 1996) as well as many other facets of the humoral immune response and clearly demonstrate that CR2 is central to the breadth of B cell function in the mouse (reviewed (Holers, 2005)).

CR2 is known to exert potent B cell signalling through non-covalent association with CD19 and CD81 (TAPA-1) (Bradbury et al., 1992; Fearon and Carter, 1995; Matsumoto et al., 1991; Tedder et al., 1994). The co-ligation of this signalling complex and the B cell receptor (BCR) are thought to form the basis of CR2s role in the humoral immune response and in the maintenance of B cell tolerance. However, the definitive roles of each of the molecules involved (antigen coated with C3d, CR2, CD19, CD81 and the BCR) remain an area of intense study and debate (reviewed (Rickert, 2005)). In order to investigate the role of CR2 in B cell function, we have previously generated several distinct hCR2 tg mice (Marchbank et al., 2002; Marchbank et al., 2000). The hCR2^high^ mice express hCR2 during the early stages of B cell development unlike endogenous mCR1/2 or hCR2 tg mice created using a hCR2 genomic fragment, (Marchbank et al., 2000) under the control of a B cell-specific lambda promoter/enhancer minigene. The hCR2^high^ mice display a 40–60% reduction in mature B cell numbers and the remaining mature B cells failed to respond adequately *in vivo* when challenged with a number of TD and TI antigens (Birrell et al., 2005; Kulik et al., 2007; Marchbank et al., 2002). We have recently found evidence of alteration in tyrosine phosphorylation patterns in response to BCR and CR2/CD19 cross-linking in the hCR2^high^ mice and demonstrated that these changes protect hCR2^high^ mice from the onset of an organ specific autoimmune disease collagen-induced arthritis (CIA) (Kulik et al., 2007).

In order to further investigate the resistance of hCR2 tg mice to onset of autoimmune disease, we crossed the hCR2^high^ mice onto the B6^lpr^ background. B6 mice do not readily develop autoimmune disease under normal circumstances (Izui et al., 1984). However, B6 mice deficient for Fas (CD95) develop a mild lupus-like disease characterized by lymphadenopathy, splenomegaly and production of increased titers of IgG antibodies to a variety of auto-antigens including DNA, anti-nuclear antigens (ANA), and the Fc portion of autologous IgG (Cohen and Eisenberg, 1991; Izui et al., 1984). Notably, B6^lpr^ mice do not develop the renal failure or arthritis associated with the deficiency in Fas in other strains of mice (Cohen and Eisenberg, 1991; Izui et al., 1984; Theofilopoulos and Dixon, 1985; Theofilopoulos et al., 1985). Herein, we show that expression of hCR2 in the B6^lpr^ mice significantly reduces the level of ANA generated and we establish that hCR2 expression on B cells in the bone marrow results in increased B cell apoptosis at the point of BCR expression, suggesting an increase in negative selection.

## 2 Materials and methods

### 2.1 Cells

Peripheral blood lymphocytes (PBL) from mice were collected into 20 μl of heparin via a tail bleed and washed once in cold PBS. Bone marrow B cells were collected by flushing mouse femurs with cold PBS. Isolated spleens were ground into single cell suspensions using frosted glass slides and transferred to 15 ml conical tubes on ice. Large debris settled after a 10 min incubation and the supernatant was transferred to a new tube. Cells were pelleted and washed once with staining buffer (PBS, 1% FBS, 0.02% sodium azide). All samples derived from mice were incubated with 0.5–1 ml of ammonium chloride red blood cell (RBC) lysis buffer at room temperature for 1–2 min. The cells were then washed with 1 ml staining buffer 1–2 times, counted and 1–3 × 10^6^ cells/ml used per analysis. Cells were then stained as described below.

### 2.2 Antibodies

Purified and biotinylated (b) 171 (anti-hCR2) and b7E9 (anti-mCR1/2), purified and bIgG_1_ (isotype control) were produced in the laboratory from hybridomas following standard methods. Purified 2.4G2 (anti-mCD16/mCD32, Fc Block), phycoerythrin (PE) conjugated B-Ly-4 (anti-hCR2), fluorescein (FITC) or allophycocynin (APC) conjugated RA3-6B2 (anti-mCD45R, B220), biotin-conjugated 145-2C11 (anti-CD3ɛ), FITC-Annexin V, Propidium Iodide (PI) and Streptavidin (SA) – APC were all obtained from Pharmingen (BD, Cowley, Oxford). SA–PE and anti-IgM-Cy5.5 were obtained from Jackson labs (Stratech Scientific,UK). CaspACE (FITC-VAD-FMK) was obtained from (Promega, Madison, WI, USA).

### 2.3 Mice

The lambda human CR2 transgenic mice (hCR2^high^ and hCR2^int^) and the genomic hCR2 transgenic mouse (P1-5) used in this study were generated and screened by PCR and/or flow cytometry as previously described (Marchbank et al., 2002; Marchbank et al., 2000). Additional mice used are F7-10 on the *Cr2*^−/−^ background (Molina et al., 1996) (confirmed by PCR or flow cytometry using b7E9), F6-F8 on the C3^−/−^ background (screened by ELISA or Western Blot as previously described (Twohig et al., 2007)), F4 on the DBA1/j (DBA) background or on a C57Bl/6j (B6) background as indicated. The hCR2^high^ mice on the C57BL/6j-Fas^lpr^/Fas^lpr^ (B6^lpr^) background were generated by back crossing for four generations whilst screening for homozygous Fas^lpr^ defect and hCR2 expression by PCR as previously described (Sekine et al., 2001; Marchbank et al., 2002) (B6^lpr.^ ^hCR2^).

### 2.4 Flow cytometry

After RBC lysis, cells were washed and resuspended in 10 μg/ml of 2.4G2 antibody in order to block Fc receptors. After 15 min incubation on ice, cells were washed in staining buffer. Cells were resuspended in 100 μl staining buffer containing primary Ab (0.1–3 μg/ml) and 1 μl anti-B220-FITC or APC, where appropriate. Cells were incubated for 30 min on ice in the dark. After incubation, cells were washed in staining buffer 3 times and then incubated with the appropriate streptavidin conjugated fluorochrome to detect biotin labeled primary Abs. Following incubation, cells were washed as above and then resuspended in staining buffer containing 1% formaldehyde. Flow cytometry was carried using a Becton-Dickinson FACS calibur (Oxford, UK).

### 2.5 Apoptosis analysis

Cells were collected from spleen or bone marrow as described above. B cells were identified using B220-APC and incubated with CaspACE according to manufacturers guidance (Promega, UK). On some occasions cells were also stained using B220-APC to identify B cells followed by PI and Annexin V FITC apoptosis kit (provided by BD Pharmingen, Oxford, UK). Samples were then immediately analysed by flow cytometer. Alternatively, cells were washed 3x in RPMI before being resuspended at 3 × 10^6^ cell/ml in RPMI/10% FBS/ 0.01% β_2_-mercaptoethanol in 24 well plates. Plates were cultured in standard conditions for 16 h. Cells were then collected and washed 3 times prior to staining as outlined above.

### 2.6 ANA, dsDNA and ssDNA ELISA

Blood was collected from age and sex matched 12 B6, 15 B6^lpr^ or 12 B6^lpr.^ ^hCR2^ mice by tail vein nick and allowed to clot. Sera was then applied to a quantitative ANA or qualitative ssDNA and dsDNA ELISA as directed by the manufacturers instructions (Autogen Bioclear,Wiltshire, UK).

### 2.7 Statistical analysis

Data was analysed and significance between groups was established using PrismGraph 3 software. Student's T test and the Mann–Whitney test were used were appropriate. *p* < 0.05 was considered significant.

## 3 Results

### 3.1 The levels of ANA are significantly reduced in B6^lpr^.hCR2^high^ mice

**Fig. 1:**
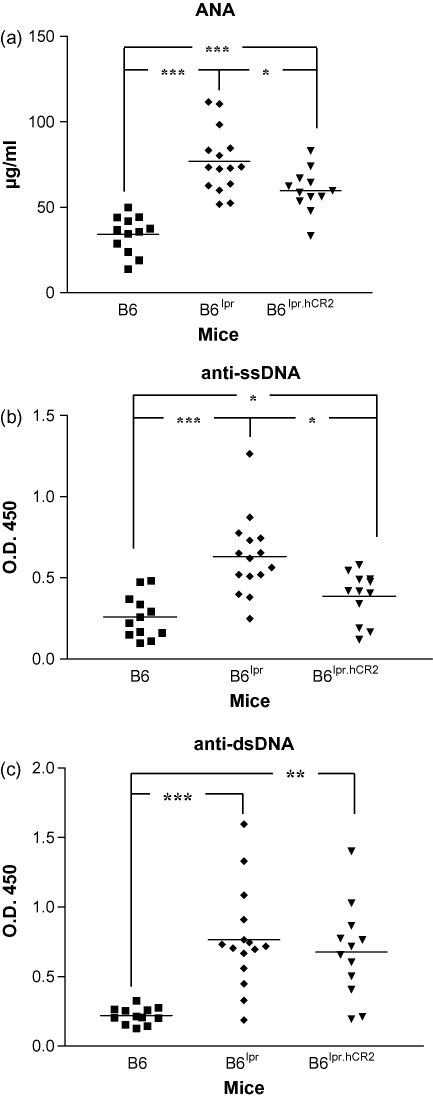
Significantly less ANA are detectable in B6^lpr^ mice which express hCR2. Serum was collected from 12 C57Bl/6j (B6), 15 C57Bl/6j-Fas^lpr^/Fas^lpr^ (B6^lpr^) and 12 B6^lpr^.hCR2^high^ (B6^lpr.hCR2^) mice at 6 month of age and applied to commercially available quantitative ANA ELISA (a) or qualitative anti-ssDNA ELISA (b) or anti-dsDNA ELISA (c) following manufacturers instructions. Significance was calculated by Students *T* test. ***p < 0.0001, **p < 0.001, *p < 0.05.

The reliance of the CIA model on adjuvant driven antibody production to produce disease (Yanaba et al., 2007) and the inability of hCR2^high^ mice to generate antibody to other model antigens (Birrell et al., 2005; Kulik et al., 2007; Twohig et al., 2007) led us to question whether the marked protection from disease seen in our previous CIA study (Kulik et al., 2007) was due to an inability to generate antibodies to the bovine collagen. To determine whether a similar effect would be seen in the absence of foreign antigen and adjuvant, we opted to test whether expression of the hCR2 tg could prevent onset of spontaneous autoimmune disease on the B6^lpr^ background. Mice were backcrossed for 4 generations and serum collected from at least 12 B6^lpr^.hCR2^high^, B6^lpr^ or B6 mice as they reached 6 months of age. The levels of ANA in B6^lpr^.hCR2^high^ mice were found to be significantly reduced compared to B6^lpr^ mice using a quantitative ANA ELISA (Fig. 1a). However, B6^lpr^.hCR2^high^ mice still generated significantly higher levels of ANA than age matched B6 mice (Fig. 1a), and there was no visible difference between these groups of mice with respect to the levels of C3 and Ig deposition in the kidney at this time (data not shown). Analysis of ssDNA and dsDNA (Fig. 1b and c) using a semi-quantitative approach confirmed significantly lower levels of ssDNA auto-antigen but only a slight decrease in dsDNA across the mice. This suggested some variation in the extent to which certain auto-antigens were affected as well as underlining that whilst expression of the hCR2 tg is clearly protective in this background, it did not give the relative degree of protection noted in our previous CIA study on the DBA1/j background.

### 3.2 B cells from hCR2^high^ mice are more susceptible to apoptosis

**Fig. 2:**
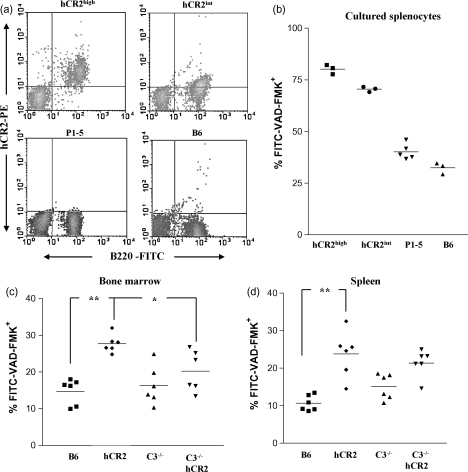
Apoptosis is increased in HCR2 tg mice. Splenocytes were isolated from littermates of each genotype as indicated in the methods section. (a) Cells were stained with B220 and b171 to confirm the expression level of hCR2 on B cells isolated from the mice indicated. (b) Cells were incubated overnight in standard tissue culture conditions and then stained with anti-B220 and FITC-VAD-FMK to identify apoptotic B cells. 3 C57Bl/6j (B6), 5 P1-5 (hCR2 tg), 3 hCR2^int^ and 3 hCR2^high^ mice were examined (each data point is the mean apoptosis value from 3 separate culture wells). (c) Freshly isolated bone marrow and (d) Splenocytes were stained with anti-B220-APC (to identify B cells) and FITC-VAD-FMK (to identify apoptotic cells). Groups of 6 mice were analysed; C57Bl/6 (B6), hCR2^high^ (hCR2); C57Bl/6.C3^−/−^(C3^−/−^), C57Bl/6.C3^−/−^.hCR2^high^ (C3^−/−^hCR2). Mann–Whitney test was used to establish significance. *p < 0.05, **p < 0.01.

Our previous studies in hCR2^high^ mice have revealed a marked change in activation marker profile of B cells in the periphery that is reminiscent of a mild anergic phenotype (Birrell et al., 2005). One marker that was elevated in hCR2^high^ mice was Fas and provides a potential explanation for the difference in protection noted between the B6^lpr^ study and the CIA study, in that the primary mechanism of protection associated with expression of hCR2 could be the removal of B cells by a Fas dependent apoptosis pathway. In order to find evidence for this possibility, we looked at the level of apoptosis in B cells isolated from hCR2^high^ mice as well as additional lines with lower hCR2 expression levels on their B cells. Prior to overnight incubation, the level of hCR2 expression on the splenic B cells was confirmed by flow cytometry (Fig. 2a) and then the levels of apoptosis displayed by the B cell populations from B6, hCR2^high^, hCR2^int^ and P1-5 mice (genomic hCR2 transgenic mouse expressing hCR2 at the correct developmental point and without the B cell defect (Marchbank et al., 2000)) were established after 16 h. The level of detectable apoptotic B cells in cultures from hCR2^high^ mice was found to be significantly greater than that of both B6 and the P1-5 strain of mice and was notably higher than hCR2^int^ mice (Fig. 2b). This finding led us to examine the baseline levels of apoptosis in freshly isolated BM and splenic B cells from hCR2^high^ mice. Again, hCR2^high^ mice displayed significantly higher levels of apoptosis than the B6 littermates (Fig. 2c and d). Examination of C3^−/−^. hCR2^high^ mice (i.e. devoid of the main ligands for CR2) suggested that C3 levels were particularly important in determining apoptosis levels during the BM stage of B cell development (Fig. 2c). Analysis of the splenic B cell population in the C3^−/−^. hCR2^high^ mice also indicated a reduced level of apoptosis but it was not nearly as marked (Fig. 2d). We found that apoptosis levels after 2 h culture were not significantly different from those measured prior to incubation but suggested that there was an increased rate of apoptosis during culture (data not shown). Overall, this data infers that expression level and/or stage of expression of hCR2 is critical in establishing the susceptibility of B cells to apoptosis in culture and might give a reason for the differences between the previous CIA study and the current B6^lpr^ analysis.

### 3.3 C3 restricts hCR2 expression levels on the B cell surface after sIgM expression

**Fig. 3:**
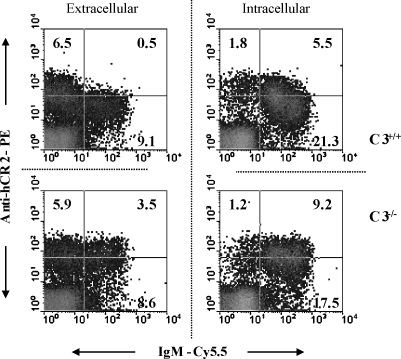
B cells from hCR2^high^ mice express less hCR2 after sIgM expression in the presence of C3. BM cells were collected from C3^+/+^hCR2^high^ and C3^−/−^hCR2^high^ mice as described in materials and methods. BM lymphocytes were stained for B220^+^ and hCR2 followed by staining for IgM before (Extracellular) or after (Intracellular) incubation with fix/perm (BD biosciences, Oxford, UK). Dot plots show B220-FITC^+^ cells expressed as hCR2-PE (Bly-4) versus anti-IgM-Cy5.5. Results shown are representative of 3 independent experiments involving 10 mice from each genotype in total.

We have previously shown that hCR2 expression levels drop significantly as the B cells mature from B220^lo^ to B220^hi^ and are reduced further still as B cells migrate into the periphery (Birrell et al., 2005). On analysis of B cells isolated from C3^−/−^ hCR2^high^ mice, we found that this reduction in hCR2 expression levels was no longer apparent, essentially leading to an apparent 3-fold increase in hCR2 expression levels on mature B cells in conjunction with an improvement in the number of B cells reaching the periphery (Twohig et al., 2007). These previous data, together with the data showing that apoptosis levels in the BM of C3^−/−^hCR2^high^ were reduced compared with hCR2^high^ mice (Fig. 2c), led us to look more closely at the factors involved in the down regulation or selection of cells with low levels of hCR2 in the presence of C3. Examination of BM B cells for intracellular and extracellular expression of sIgM, in conjunction with hCR2 surface expression, demonstrated that sIgM expressing cells had lower levels of hCR2 on the cell surface than B cells which had constructed but not expressed sIgM (Fig. 3). These results were confirmed using several different antibodies to hCR2 in order to control for the potential loss or blocking of antibody binding sites due to the presence and binding of newly formed C3 fragments to CR2 in the C3 sufficient mice (data not shown). When this analysis was subsequently carried out in the C3^−/−^ background, we found that hCR2 expression levels on cells that expressed sIgM were not significantly different from those before sIgM was expressed. Overall, these data suggest that the levels of hCR2 are tightly regulated on the mouse B cell at the point of sIgM expression and that C3 and/or its break down components play an important part in this mechanism.

### 3.4 Background strain influences the degree of B cell reduction in hCR2 tg mice

**Fig. 4:**
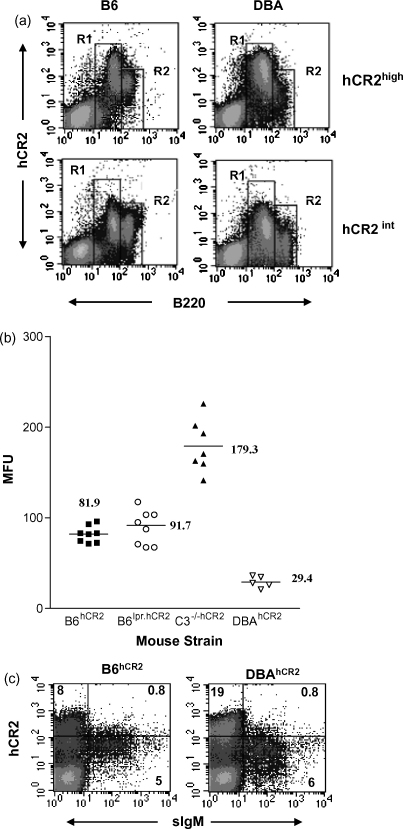
hCR2 expression level is dependent on mouse strain. (a) Representative plots of BM cells isolated from age and sex matched hCR2^high^ and hCR2^int^ mice on the DBA/1j and C57Bl/6 background are shown. B220-FITC was used to identify B cells and hCR2 expression was assessed using biotin-171 + SA-PE. R1 and R2 delineate the B220^low^ (pre/immature) and B220^high^ (mature) B cell population, respectively. (b) Splenocytes from 8 C57Bl/6.hCR2^high^ (B6^hCR2^), 9 B6^lpr^.hCR2^high^ (B6^lpr.hCR2^), 7 C57Bl/6.C3^−/−^.hCR2^high^ (C3^−/−hCR2^) and 5 DBA/1j.hCR2^high^ (DBA^hCR2^) were stained with B220 and b171 to establish the relative level of hCR2 expression on mature B cells in these strains. The mean value of each group is shown next to each group and illustrated by the horizontal bar. (c) BM cells were isolated from age and sex matched hCR2^high^ mice on the B6 and DBA background. Dot plots show B220-FITC^+^ cells expressed as hCR2 (biotin 171 + SA-PE) versus anti-IgM-Cy5.5 (extracellular). 10,000 B cells were collected by FACS and results are representative of at least 5 mice analysed for each strain.

During our recent study using CIA, where the hCR2 tg was backcrossed onto the DBA1/j background (Kulik et al., 2007), it quickly became apparent that hCR2 expression levels on mature splenic B cells isolated from both hCR2^high^ and hCR2^int^ mice were at low levels as determined by flow cytometry analysis. Considering the data from above, we studied hCR2 expression levels during B cell development in the DBA.hCR2^high^ mice in comparison to our standard B6 strain. We found that the expression level of hCR2 on the B220^lo^ B cell population of the bone marrow in both hCR2^high^ and hCR2^int^ mice was almost identical between the B6 and DBA strains of mice (Fig. 4a, R1). However, as the B cells matured (become B220^high^), B cell surface expression of hCR2 was found to be significantly lower in the DBA background than that seen in B6 background (Fig. 4a, R2). This was even more apparent in the mice expressing the intermediate level of hCR2^int^, where the reduction in hCR2 expression is minimal in the B6 strain but well defined in the DBA background. When this analysis was extended to include hCR2^high^ mice on the B6^lpr^ background, we found that there was no real change in hCR2 expression levels on mature B cells between the B6 and B6^lpr^ backgrounds (Fig. 4b). As previously noted, these data are in contrast to hCR2^high^ mice devoid of C3 (B6.C3^−/−^) which have a marked increase in hCR2 expression levels on mature B cells compared with their wild type B6 counterparts (Fig. 4b, Twohig et al., 2007). Analysis of surface IgM and hCR2 expression revealed that the reduction in B cell expression of hCR2 in the DBA background appeared to coincide with the expression of sIgM (Fig. 4c). This data demonstrates that strain differences exist in respect to the extent to which hCR2 is considered ‘excessive’ on the B cell surface during this stage of B cell development. It also indicates that a common mechanism is likely to exist between these two strains of mice and that expression of sIgM triggers cell death and the modulation of hCR2 expression levels seen on mature B cells.

### 3.5 DBA.hCR2^high^ mice have significantly fewer B cells than B6^lpr^ or B6 backgrounds

**Fig. 5:**
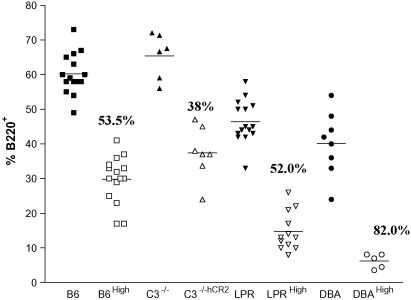
Peripheral B cell percentages are dependent on background strain. Blood from mice was collected into 20 μl of heparin and red cells lysed. B cells and T cells were further identified by B220 and CD3, respectively. Fixed samples were then assessed by flow cytometry. PBL were identified by their forward and side scatter profile and the relative percentage of B220 positive cells falling in the lymphoid gate was calculated for each strain of mouse as indicated. 15 C57Bl/6 (B6), 15 C57Bl/6.hCR2^high^ (B6^hCR2^), 6 C57Bl/6.C3^−/−^ (C3^−/−^), 7 C57Bl/6.C3^−/−^.hCR2^high^ (C3^−/−hCR2^), 15 C57Bl/6j- Fas^lpr^/Fas^lpr^ (B6^lpr^), 12 B6^lpr^.hCR2^high^ (B6^lpr^^hCR2^), 8 DBA/1j (DBA) and 5 DBA/1j .hCR2^high^ (DBA^hCR2^) were analysed. Wild type mice (hCR2 negative) are represented by the filled-in symbols for each strain and the open symbols represent mice that express hCR2. The mean value for the percentage B cells found in the blood of each group of mice is illustrated by the horizontal line. The reduction in B cell percentages (from that normally seen in the hCR2 negative littermates) is shown directly above the hCR2^high^ expressing groups.

The first characteristic to be described in the lambda hCR2 transgenic mice is the loss of mature B cells from blood. Analysis of PBL percentages suggested that up to 60% of normal B cells are deleted in hCR2^high^ compared with wild type B6 littermates (Marchbank et al., 2002). Analysis of B cell percentages in B6, B6^lpr^ and DBA strains indicated that these mice display different intrinsic B cell levels in the blood with B6 having the highest percentages followed by B6^lpr^ and DBA, respectively (Fig. 5). Despite our initial thoughts, that there may be a reduction in apoptosis on the B6^lpr.^hCR2^high^ mice, it was clear that peripheral B cell numbers were reduced in a similar manner in the B6^lpr^ background as seen in the B6 background (B6^lpr^.hCR2^high^ 52% to hCR2^high^ 53% of normal). However, the expression of hCR2 on B cells in the DBA background results in a further significant reduction in peripheral B cells. In fact, B cells are severely reduced in the DBA.hCR2^high^ mice, with the percentage of B cells in the lymphoid gate dropping to less than 20% of normal (or around 7% of total lymphocytes in the blood compared with nearly 40% in the hCR2 negative DBA littermates).

## 4 Discussion

Most autoimmune disorders are characterized by the presence of auto-antibodies and abnormalities of B-cell function (Browning, 2006; Martin and Chan, 2006). However, the mechanism(s) that leads to break down of tolerance in B cells remains elusive. There is a growing body of evidence that CR1/CR2 plays an important role in maintaining and defining normal B cell function and thereby helps to prevent the onset of autoimmune disease (reviewed (Holers, 2005)). Our own previous findings demonstrated that the hCR2^high^ mice were almost completely protected from organ specific autoimmune disease (using the CIA model) (Kulik et al., 2007) and raised our hopes that these mice were largely protected from all autoimmune diseases.

These hopes were partially born out, in that B6^lpr.^hCR2^high^ were found to produce significantly lower levels of ANA than B6^lpr^ mice that did not express hCR2 (Fig. 1a). However, the levels of ANA were not reduced back to the levels found in the B6 strain (which are largely refractory to spontaneous ANA generation (Izui et al., 1984; Theofilopoulos and Dixon, 1985; Theofilopoulos et al., 1985) and the data indicated that significant levels of auto-antibodies still accumulate in the B6^lpr^.hCR2^high^ mice. This was confirmed, albeit in a qualitative rather than a quantitative anti-ssDNA ELISA (Fig. 1b). Anti-dsDNA are generally accepted as one of the defining features of the MRL^lpr^ model (Theofilopoulos and Dixon, 1985; Theofilopoulos et al., 1985), but this analysis demonstrated that the number of animals with readily detectable anti-dsDNA reactivity was not significantly different between the hCR2 expressing and non-expressing B6^lpr^ littermates. The difference noted between dsDNA and ANA results might be a result of the sensitivity of these different assays or may reflect a selective deletion of particular BCR specificities during B cell development in the presence of hCR2. Analysis of deposited C3 and Ig in several randomly selected kidneys from B6^lpr^ and B6^lpr^.hCR2^high^ mice (data not shown) also confirmed that the protective effect of hCR2 expression was less obvious than that seen in the CIA model (Kulik et al., 2007). Significant increases in ANA and rheumatoid factor titres have been found in B6^lpr^ and MRL^lpr^ mice in the absence of CR2 but these have little negative effect with respect to renal function (Boackle et al., 2004; Wu et al., 2002) and suggests that whilst CR2 is highly important with respect to the type, level and specificity of auto-antibodies generated in the absence of Fas, it does not dramatically alter the outcome of this autoimmune disease model. Due to a limited amount of sera remaining after the initial analysis, we were unable to establish the effect of hCR2 on other auto-antibodies such as rheumatoid factor but the data herein suggests that certain B cell clones maybe lost in the B6^lpr^.hCR2^high^ mice that are at least partially beneficial with respect to development and accumulation of ANA. However, as might be predicted from the CR2 knockout studies mentioned above (Boackle et al., 2004; Wu et al., 2002), the presence of hCR2 is not sufficient to completely reverse the autoimmune phenotype in this background.

Analysis of the percentage of B cells in the peripheral blood lymphocyte compartment suggest that there is a significantly higher level of B cell deletion in the DBA background (albeit in a smaller experimental group) than in the B6^lpr^ background (Fig. 5). These data suggest that lack of available B cells in the DBA mice compared to those available in the B6^lpr^ mice could largely explain the difference in hCR2s ability to protect against disease onset. A recent study by Yanaba et al. (2007), where B cells were depleted using anti-CD20 antibodies prior to running the CIA model, clearly demonstrated the importance of B cells in driving disease in the CIA model. Interestingly, the level of B cell deletion (approximately 95%) and the initial outcomes of B cell deletion with anti-CD20 is highly similar to that noted in the DBA.hCR2^high^ mice. These data continue to support the idea that B cells play a significant role in the pathogenesis of CIA and considering B cell numbers are not reduced as significantly in the B6^lpr^.hCR2^high^ background compared to the DBA.hCR2^high^ background (Fig. 5), offer a very plausible explanation for the difference in the level of protection noted in CIA on the DBA background versus the effects noted in B6^lpr^ model.

The failure of “anergic-like” hCR2^high^ B cells (Birrell et al., 2005) to protect from onset of disease in the B6^lpr^ model might fit with the fact that B cells contribute more than just auto-antibodies in the autoimmune response in lupus-prone models, i.e. B cells have an important APC role in these mice (Chan et al., 1997; Chan et al., 1999). As T cell responses were found to be essentially normal in the previous CIA experiment (on the DBA background) (Kulik et al., 2007), it is probably fair to assume that the APC function of B cells or DC remain intact in the B6^lpr^ mice. Thus, in the B6^lpr^ model the ability of T cells to drive B cells to become autoreactive should remain fully intact and suggests that the loss of B cell numbers and the alteration of signalling pathways in the remaining B cell population appears to be overcome in the B6^lpr^ mice through autoreactive T cell help. Together, these data support the notion that the MRL model is primarily reliant on misdirected T cell function/tolerance whilst the CIA model is primarily reliant on misdirected B cell function/tolerance and thus effects from altering signal pathways via BCR and CR2 cross-linking are more likely to have a defined impact in the CIA model.

We have previously postulated that expression of hCR2 in the bone marrow influences the signal through the Pre-BCR (Kulik et al., 2007), which is normally required to allow B cells to progress along the B cell maturation pathway (Rolink and Melchers, 1996). Analysis of apoptosis levels in the hCR2^high^ mice established that they had an increased basal rate of apoptosis in both the bone marrow and spleen (Fig. 2). This data suggests that B cells in the hCR2^high^ mice are highly susceptible to apoptosis, particularly at the point of surface IgM expression. The presence of C3 (Fig. 3) appears to be linked directly to the reduction of CR2 expression level on the B cell surface (Fig. 4a) and suggests that hCR2, interacting with C3 and the sIgM/BCR is interfering with the normal negative selection process. This could be a result of hCR2 modulating the level of signal perceived from the sIgM. In order to investigate this assertion, we are currently crossbreeding the hCR2^high^ line with mice expressing only BCR with specificity for chicken lysozyme (HEL tg mice (Goodnow et al., 1988)).

The fact that genetic background and the selection of the naïve B cell repertoire have been linked to the susceptibility of mice to autoimmune disease (Wang et al., 2003) supports the idea that hCR2 can amplify normal selective pressure in this model. Certainly B6 and the B6^lpr^ strain have a marked decrease in B cells transiting the BM compared with hCR2 negative littermates but surprisingly the DBA mice have the fewest surviving B cells in the periphery (Fig. 5) and suggests that they have a highly restricted B cell repertoire which is being further tightened in the presence of hCR2. Direct analysis of B cell susceptibility to altered deletion in B6^lpr^ might be informative. However, it has already been established that deletion of autoreactive B cells after BCR ligation occurs unabated in the absence of Fas (Yoshida et al., 2000) and we found that the levels of B cells in the B6^lpr^.hCR2 mice were essentially identical to those seen in B6.hCR2 mice (Fig. 5). Thus, as the effects of hCR2 expression appear to be exerted on the BCR signalling pathways it seems unlikely that significant differences would be seen between the hCR2^high^ and B6^lpr^.hCR2^high^ mice.

Corroborating evidence for enhanced BCR signalling being the likely mechanism behind the altered phenotype in the hCR2^high^ mice can be found in the altered patterns of tyrosine phosphorylated proteins in B cells from hCR2 tg mice before and after BCR cross-linking (Kulik et al., 2007). Interestingly, mobilization of Ca^2+^ in immature B cells has been found to be much greater than that seen in mature B cells, presumably to amplify weak signals in the BM environment and delete autoreactive B cells from the repertoire (Benschop et al., 2001). It thus seems plausible that additional signal amplification via hCR2 in the BM is likely to disrupt these highly sensitive signalling networks resulting in the higher levels of apoptosis found and the increase in anergy in the remaining B cells.

The data presented here continues to suggest that CR2 expression levels are a critical factor in determining B cell function. The signal generated through hCR2 is apparently a modifier of the BCR signal since numbers of pro-B cells and hCR2 expression levels are ‘normal’ until surface BCR is expressed in the BM. It is possible that additional signalling molecules expressed at the same point as sIgM may interact with hCR2 and thus sIgM might only be a marker of the time when such changes take place. Nevertheless, it is clear that examination of the proteins and signalling molecules being altered in the hCR2^high^ mice at the point that of BCR, hCR2 and (self) antigen contact will provide a fuller understanding of the molecules involved in B cell survival and selection of the BCR repertoire. This should also provide a greater understanding of the mechanisms that predispose B cells to break tolerance in certain genetic backgrounds.

## References

[bib1] Ahearn J.M., Fearon D.T. (1989). Structure and function of the complement receptors, CR1 (CD35) and CR2 (CD21). Adv. Immunol..

[bib2] Ahearn J.M., Fischer M.B., Croix D., Goerg S., Ma M., Xia J., Zhou X., Howard R.G., Rothstein T.L., Carroll M.C. (1996). Disruption of the Cr2 locus results in a reduction in B-1a cells and in an impaired B cell response to T-dependent antigen. Immunity.

[bib3] Benschop R.J., Brandl E., Chan A.C., Cambier J.C. (2001). Unique signalling properties of B cell antigen receptor in mature and immature B cells: implications for tolerance and activation. J. Immunol..

[bib4] Birrell L., Kulik L., Morgan B.P., Holers V.M., Marchbank K.J. (2005). B cells from mice prematurely expressing human complement receptor type 2 are unresponsive to T-dependent antigens. J. Immunol..

[bib5] Boackle S.A., Culhane K.K., Brown J.M., Haas M., Bao L., Quigg R.J., Holers V.M. (2004). CR1/CR2 deficiency alters IgG3 autoantibody production and IgA glomerular deposition in the MRL/lpr model of SLE. Autoimmunity.

[bib6] Boackle S.A., Holers V.M., Chen X., Szakonyi G., Karp D.R., Wakeland E.K., Morel L. (2001). Cr2, a candidate gene in the murine sle1c lupus susceptibility locus, encodes a dysfunctional protein. Immunity.

[bib7] Bradbury L.E., Kansas G.S., Levy S., Evans R.L., Tedder T.F. (1992). The CD19/CD21 signal transducing complex of human B lymphocytes includes the target of antiproliferative antibody-1 and Leu-13 molecules. J. Immunol..

[bib8] Browning J.L. (2006). B cells move to centre stage: novel opportunities for autoimmune disease treatment. Nat. Rev. Drug Discov..

[bib9] Carroll M.C. (2000). The role of complement in B cell activation and tolerance. Adv. Immunol..

[bib10] Chan O., Madaio M.P., Shlomchik M.J. (1997). The roles of B cells in MRL/lpr murine lupus. Ann. N. Y. Acad. Sci..

[bib11] Chan O.T., Hannum L.G., Haberman A.M., Madaio M.P., Shlomchik M.J. (1999). A novel mouse with B cells but lacking serum antibody reveals an antibody-independent role for B cells in murine lupus. J Exp. Med..

[bib12] Cohen P.L., Eisenberg R.A. (1991). Lpr and gld: single gene models of systemic autoimmunity and lymphoproliferative disease. Annu. Rev. Immunol..

[bib13] Fearon D.T., Carter R.H. (1995). The CD19/CR2/TAPA-1 complex of B lymphocytes: linking natural to acquired immunity. Annu. Rev. Immunol..

[bib14] Fingeroth J.D. (1990). Comparative structure and evolution of murine CR2. The homolog of the human C3d/EBV receptor (CD21). J. Immunol..

[bib15] Fischer M.B., Ma M., Goerg S., Zhou X., Xia J., Finco O., Han S., Kelsoe G., Howard R.G., Rothstein T.L., Kremmer E., Rosen F.S., Carroll M.C. (1996). Regulation of the B cell response to T-dependent antigens by classical pathway complement. J. Immunol..

[bib16] Goodnow C.C., Crosbie J., Adelstein S., Lavoie T.B., Smith-Gill S.J., Brink R.A., Pritchard-Briscoe H., Wotherspoon J.S., Loblay R.H., Raphael K. (1988). Altered immunoglobulin expression and functional silencing of self-reactive B lymphocytes in transgenic mice. Nature.

[bib17] Gustavsson S., Kinoshita T., Heyman B. (1995). Antibodies to murine complement receptor 1 and 2 can inhibit the antibody response *in vivo* without inhibiting T helper cell induction. J. Immunol..

[bib18] Haas K.M., Hasegawa M., Steeber D.A., Poe J.C., Zabel M.D., Bock C.B., Karp D.R., Briles D.E., Weis J.H., Tedder T.F. (2002). Complement receptors CD21/35 link innate and protective immunity during Streptococcus pneumoniae infection by regulating IgG3 antibody responses. Immunity.

[bib19] Hebell T., Ahearn J.M., Fearon D.T. (1991). Suppression of the immune response by a soluble complement receptor of B lymphocytes. Science.

[bib20] Heyman B., Wiersma E.J., Kinoshita T. (1990). *In vivo* inhibition of the antibody response by a complement receptor-specific monoclonal antibody. J. Exp. Med..

[bib21] Holers V.M. (2005). Complement receptors and the shaping of the natural antibody repertoire. Springer Semin. Immunopathol..

[bib22] Izui S., Kelley V.E., Masuda K., Yoshida H., Roths J.B., Murphy E.D. (1984). Induction of various autoantibodies by mutant gene lpr in several strains of mice. J. Immunol..

[bib23] Kulik L., Marchbank K.J., Lyubchenko T., Kuhn K.A., Liubchenko G.A., Haluszczak C., Gibson M.G., Boackle S.A., Holers V.M. (2007). Intrinsic B cell hypo-responsiveness in mice prematurely expressing human CR2/CD21 during B cell development. Eur. J. Immunol..

[bib24] Kurtz C.B., O’Toole E., Christensen S.M., Weis J.H. (1990). The murine complement receptor gene family. IV. Alternative splicing of Cr2 gene transcripts predicts two distinct gene products that share homologous domains with both human CR2 and CR1. J. Immunol..

[bib25] Marchbank K.J., Kulik L., Gipson M.G., Morgan B.P., Holers V.M. (2002). Expression of human complement receptor type 2 (CD21) in mice during early B cell development results in a reduction in mature B cells and hypogammaglobulinemia. J. Immunol..

[bib26] Marchbank K.J., Watson C.C., Ritsema D.F., Holers V.M. (2000). Expression of human complement receptor 2 (CR2, CD21) in Cr2^−/−^ mice restores humoral immune function. J. Immunol..

[bib27] Marquart H.V., Svendsen A., Rasmussen J.M., Nielsen C.H., Junker P., Svehag S.E., Leslie R.G. (1995). Complement receptor expression and activation of the complement cascade on B lymphocytes from patients with systemic lupus erythematosus (SLE). Clin. Exp. Immunol..

[bib28] Martin F., Chan A.C. (2006). B cell immunobiology in disease: evolving concepts from the clinic. Annu. Rev. Immunol..

[bib29] Matsumoto A.K., Kopicky-Burd J., Carter R.H., Tuveson D.A., Tedder T.F., Fearon D.T. (1991). Intersection of the complement and immune systems: a signal transduction complex of the B lymphocyte-containing complement receptor type 2 and CD19. J. Exp. Med..

[bib30] Molina H., Holers V.M., Li B., Fung Y., Mariathasan S., Goellner J., Strauss-Schoenberger J., Karr R.W., Chaplin D.D. (1996). Markedly impaired humoral immune response in mice deficient in complement receptors 1 and 2. Proc. Natl. Acad. Sci. U.S.A..

[bib31] Molina H., Kinoshita T., Inoue K., Carel J.C., Holers V.M. (1990). A molecular and immunochemical characterization of mouse CR2. Evidence for a single gene model of mouse complement receptors 1 and 2. J. Immunol..

[bib32] Moore M.D., Cooper N.R., Tack B.F., Nemerow G.R. (1987). Molecular cloning of the cDNA encoding the Epstein–Barr virus/C3d receptor (complement receptor type 2) of human B lymphocytes. Proc. Natl. Acad. Sci. U.S.A..

[bib33] Prodeus A.P., Goerg S., Shen L.M., Pozdnyakova O.O., Chu L., Alicot E.M., Goodnow C.C., Carroll M.C. (1998). A critical role for complement in maintenance of self-tolerance. Immunity.

[bib34] Rickert R.C. (2005). Regulation of B lymphocyte activation by complement C3 and the B cell coreceptor complex. Curr. Opin. Immunol..

[bib35] Rolink A., Melchers F. (1996). B-cell development in the mouse. Immunol. Lett..

[bib36] Sekine H., Reilly C.M., Molano I.D., Garnier G., Circolo A., Ruiz P., Holers V.M., Boackle S.A., Gilkeson G.S. (2001). Complement component C3 is not required for full expression of immune complex glomerulonephritis in MRL/lpr mice. J. Immunol..

[bib37] Takahashi K., Kozono Y., Waldschmidt T.J., Berthiaume D., Quigg R.J., Baron A., Holers V.M. (1997). Mouse complement receptors type 1 (CR1;CD35) and type 2 (CR2;CD21): expression on normal B cell subpopulations and decreased levels during the development of autoimmunity in MRL/lpr mice. J. Immunol..

[bib38] Tedder T.F., Clement L.T., Cooper M.D. (1984). Expression of C3d receptors during human B cell differentiation: immunofluorescence analysis with the HB-5 monoclonal antibody. J. Immunol..

[bib39] Tedder T.F., Zhou L.J., Engel P. (1994). The CD19/CD21 signal transduction complex of B lymphocytes. Immunol. Today.

[bib40] Theofilopoulos A.N., Dixon F.J. (1985). Murine models of systemic lupus erythematosus. Adv. Immunol..

[bib41] Theofilopoulos A.N., Prud’Homme G.J., Dixon F.J. (1985). Autoimmune aspects of systemic lupus erythematosus. Concepts Immunopathol..

[bib42] Thyphronitis G., Kinoshita T., Inoue K., Schweinle J.E., Tsokos G.C., Metcalf E.S., Finkelman F.D., Balow J.E. (1991). Modulation of mouse complement receptors 1 and 2 suppresses antibody responses *in vivo*. J. Immunol..

[bib43] Twohig J., Kulik L., Haluszczak C., Reuter J., Rossbach A., Bull M., Holers V.M., Marchbank K.J. (2007). Defective B cell ontogeny and immune response in human complement receptor 2 (CR2, CD21) transgenic mice is partially recovered in the absence of C3. Mol. Immunol..

[bib44] Wang C., Khalil M., Ravetch J., Diamond B. (2003). The naive B cell repertoire predisposes to antigen-induced systemic lupus erythematosus. J. Immunol..

[bib45] Weis J.J., Toothaker L.E., Smith J.A., Weis J.H., Fearon D.T. (1988). Structure of the human B lymphocyte receptor for C3d and the Epstein–Barr virus and relatedness to other members of the family of C3/C4 binding proteins. J. Exp. Med..

[bib46] Wilson J.G., Ratnoff W.D., Schur P.H., Fearon D.T. (1986). Decreased expression of the C3b/C4b receptor (CR1) and the C3d receptor (CR2) on B lymphocytes and of CR1 on neutrophils of patients with systemic lupus erythematosus. Arthritis Rheum..

[bib47] Wu H., Boackle S.A., Hanvivadhanakul P., Ulgiati D., Grossman J.M., Lee Y., Shen N., Abraham L.J., Mercer T.R., Park E., Hebert L.A., Rovin B.H., Birmingham D.J., Chang D.M., Chen C.J., McCurdy D., Badsha H.M., Thong B.Y., Chng H.H., Arnett F.C., Wallace D.J., Yu C.Y., Hahn B.H., Cantor R.M., Tsao B.P. (2007). Association of a common complement receptor 2 haplotype with increased risk of systemic lupus erythematosus. Proc. Natl. Acad. Sci. U.S.A..

[bib48] Wu X., Jiang N., Deppong C., Singh J., Dolecki G., Mao D., Morel L., Molina H.D. (2002). A role for the Cr2 gene in modifying autoantibody production in systemic lupus erythematosus. J. Immunol..

[bib49] Yanaba K., Hamaguchi Y., Venturi G.M., Steeber D.A., St Clair E.W., Tedder T.F. (2007). B cell depletion delays collagen-induced arthritis in mice: arthritis induction requires synergy between humoral and cell-mediated immunity. J. Immunol..

[bib50] Yoshida T., Higuchi T., Hagiyama H., Strasser A., Nishioka K., Tsubata T. (2000). Rapid B cell apoptosis induced by antigen receptor ligation does not require Fas (CD95/APO-1), the adaptor protein FADD/MORT1 or CrmA-sensitive caspases but is defective in both MRL-+/+ and MRL-lpr/lpr mice. Int. Immunol..

